# Prenatal and Neonatal Pulmonary Thrombosis as a Potential Complication of SARS-CoV-2 Infection in Late Pregnancy

**DOI:** 10.3390/ijms24087629

**Published:** 2023-04-21

**Authors:** Gazala Abdulaziz-Opiela, Anna Sobieraj, Greta Sibrecht, Julia Bajdor, Bartłomiej Mroziński, Zuzanna Kozłowska, Rafał Iciek, Katarzyna Wróblewska-Seniuk, Ewa Wender-Ożegowska, Tomasz Szczapa

**Affiliations:** 1 Faculty of Medicine, Poznan University of Medical Sciences, 61-701 Poznan, Poland; 2 II Department of Neonatology, Poznan University of Medical Sciences, 61-701 Poznan, Poland; 3 Department of Radiology, Nicolaus Copernicus Hospital, 80-803 Gdansk, Poland; 4 Department of Pediatric Cardiology, Poznan University of Medical Sciences, 61-701 Poznan, Poland; 5 Department of Reproduction, Poznan University of Medical Sciences, 61-701 Poznan, Poland

**Keywords:** pulmonary thrombosis, SARS-CoV-2, neonatal thrombosis, COVID-19 complications

## Abstract

Neonatal venous thrombosis is a rare condition that can be iatrogenic or occur due to viral infections or genetic mutations. Thromboembolic complications are also commonly observed as a result of SARS-CoV-2 infections. They can affect pediatric patients, especially the ones suffering from multisystem inflammatory syndrome in children (MIS-C) or multisystem inflammatory syndrome in neonates (MIS-N). The question remains whether the maternal SARS-CoV-2 infection during pregnancy can lead to thromboembolic complications in fetuses and neonates. We report on a patient born with an embolism in the arterial duct, left pulmonary artery, and pulmonary trunk, who presented several characteristic features of MIS-N, suspecting that the cause might have been the maternal SARS-CoV2 infection in late pregnancy. Multiple genetic and laboratory tests were performed. The neonate presented only with a positive result of IgG antibodies against SARS-CoV-2. He was treated with low molecular weight heparin. Subsequent echocardiographic tests showed that the embolism dissolved. More research is necessary to evaluate the possible neonatal complications of maternal SARS-CoV-2 infection.

## 1. Introduction

Neonatal venous thrombosis is a rare condition that occurs most often in infants born between the 22nd and 27th week of pregnancy [1]. Up to 90% of venous thromboembolisms are iatrogenic and are associated with central venous catheters [2,3]. Other predisposing factors are mechanical ventilation, infections with cardiotropic viruses (e.g., parvovirus B19, influenza virus, human immunodeficiency virus, cytomegalovirus, herpes simplex virus) [4], hospital stays equal or longer than five days [5], and genetic mutations (e.g., Factor V, Factor II, methylenetetrahydrofolate reductase (MTHFR) genes, protein S or C deficiencies) [6].

While SARS-CoV-2 infection most often leads to respiratory disease, it must be acknowledged that the virus might affect other systems and organs as well. The non-respiratory complications of SARS-CoV-2 infection such as preeclampsia [7] or neurological diseases [8] have been described in the literature. Thromboembolic complications are commonly observed due to SARS-CoV-2 infections, especially among adults [9]. They can also affect pediatric patients, particularly those suffering from the multisystem inflammatory syndrome in children (MIS-C) [10]. The hypothesis of maternal infection playing the pathophysiological role in neonatal thrombosis development has already been described in the literature [11]. As SARS-CoV-2 can be transmitted through the placenta [12], the neonate can develop multisystem inflammatory syndrome in neonates (MIS-N) after birth due to maternal infection [13]. We present a case of a neonate born with a pulmonary embolism in the arterial duct, left pulmonary artery, and pulmonary trunk and several characteristic features of MIS-N potentially associated with the maternal SARS-CoV-2 infection.

## 2. Case Presentation

The mother, in the 40th week of pregnancy, was admitted to the hospital with no uterine contractions for observation before labor. She reported having had an infection of probable viral etiology in the 38th week of pregnancy with fever, headache, fatigue, and intense cough. The disease happened during the COVID-19 pandemic, while the number of daily new cases was reaching peak values. Despite the symptoms of SARS-CoV-2 infection, the mother did not perform a test. She has not been vaccinated against SARS-CoV-2 either. A fetal ultrasound on admission showed an enlarged heart, asymmetrical atria, and fluid in the pericardium and abdomen cavity. Previous ultrasound scans did not show such abnormalities. Due to the suspicion of circulatory failure, the patient was transferred to a third-level referral hospital, and a cesarean section was performed.

The neonate was hypotrophic (<3rd percentile), with a birth weight of 2580 g. However, there was no evidence of fetal growth restriction in ultrasound scans performed in the 3rd trimester. The Apgar score was 8 in the 1st and 10 in the 5th minute of life. For the first five minutes of life, he required Continuous Positive Airway Pressure (CPAP) respiratory support with a maximal FiO_2_ of 25%. Blood samples from the umbilical cord were collected, and pH values from umbilical vessels were within the normal range (pH 7.31 and 7.36, BE: −0.65 and −1.81, respectively). During the first and second days of life, he had recurrent desaturations and required constant passive oxygen therapy with FiO_2_ between 25 and 30%.

**Figure 1:**
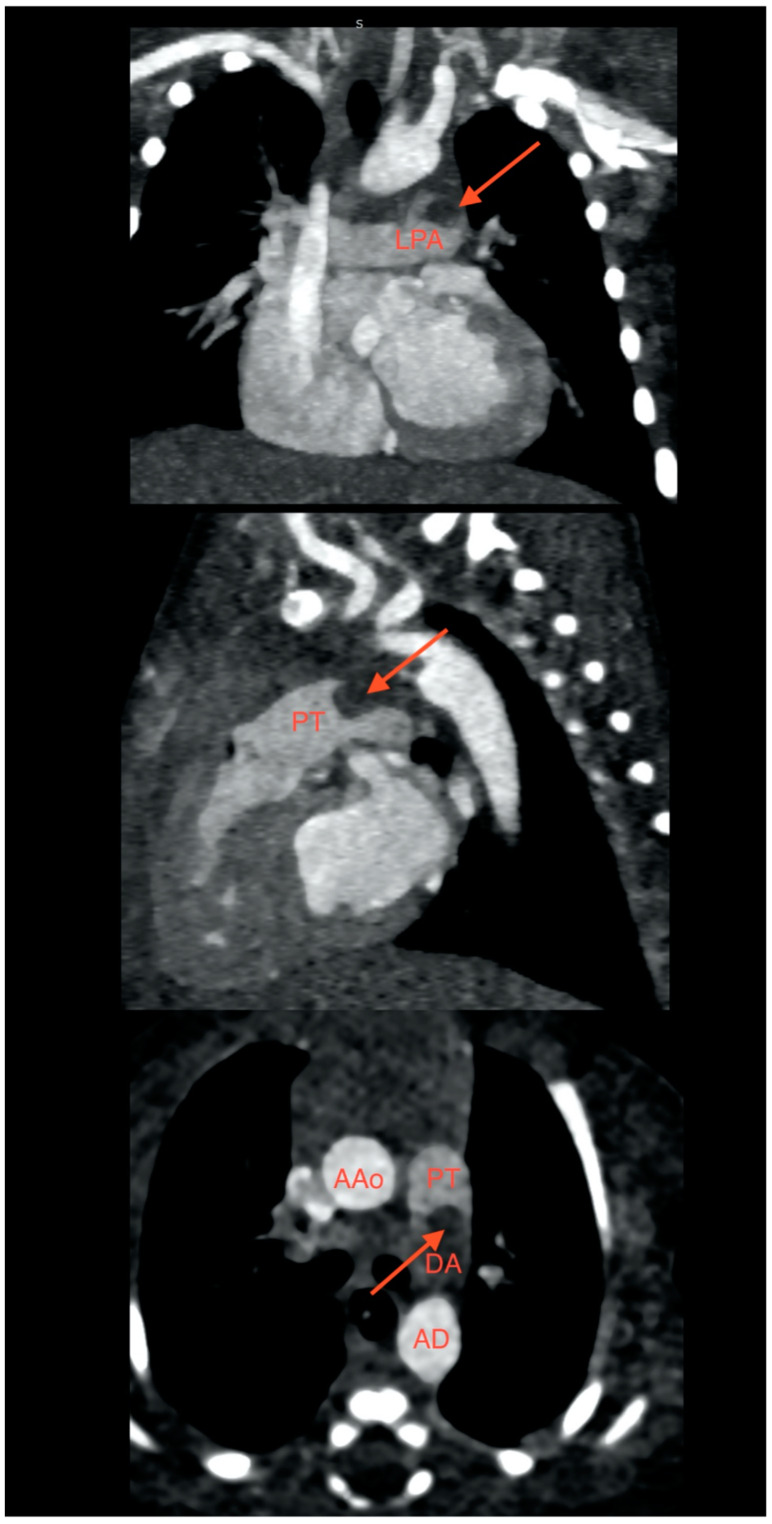
Angio-CT scan of 4-day-old neonate presenting thrombus extending into the left pulmonary artery, pulmonary trunk, and arterial duct. LPA: Left pulmonary artery; PT: Pulmonary trunk; DA: Arterial duct; AAo: Ascending aorta; AD: Descending aorta.

Echocardiography was performed twice during the initial hospital stay—in the 1st and 12th hour of life. It revealed enlarged heart atria and a spherical structure with a diameter of 4.5 mm at the connection point between the arterial duct and the left pulmonary artery. Moreover, the right ventricle’s systolic dysfunction was observed. To confirm the presence of the suspected pulmonary embolism, on the 3rd day of life, chest computed tomography angiography (chest angio-CT) was performed (Figure 1), which demonstrated the presence of an embolism located in the arterial duct, left pulmonary artery, and pulmonary trunk (size 15 mm × 4.5 mm × 4.5 mm). In the cross-section image, the thrombus occupied more than half of the lumen of the pulmonary trunk and narrowed the blood inflow to the left pulmonary artery.

The abdominal ultrasonography performed on the first day of life showed an enlarged liver and free peritoneal fluid with no other abnormalities. The additional laboratory tests in the neonate suggested an abnormal liver function (ALT: 172 IU/L, AST: 178 IU/L) and normalized with time. The albumin level was initially low (2.51 g/dL) and increased later (3.31 g/dL). The initial C-reactive protein level was 11.53 mg/L and decreased to 2.5 mg/L on the 9th day of life. Cranial ultrasound was performed twice (on the 6th and 12th day of life) and showed higher echogenicity of white matter along lateral ventricles. A follow-up ultrasound was recommended on an outpatient basis.

Multiple genetic tests were performed to find the cause of embolism formation, such as factor II, factor V, the MTHFR gene, and PAI-1 gene. The patient only tested heterozygous in the MTHFR C677T and A1298C polymorphisms and positive in the PAI-1 5G/4G polymorphism. The results of the remaining tests were normal. The biological mother had not had any medical history of thrombotic diseases, nor other members of the neonate’s family.

Moreover, laboratory tests were performed for infections with cardiotropic viruses, ruling out cytomegalovirus, adenovirus, parvovirus-B19, enteroviruses, Coxsackie B viruses, human herpes virus 6, and influenza A and B virus infections. Furthermore, a reverse transcription polymerase chain reaction (RT-PCR) test and an antibodies test for SARS-CoV-2 were performed. The neonate presented with a positive result of IgG antibodies against SARS-CoV-2.

In coagulation tests, we observed increased D-Dimers levels (7.42 mg/L), standard prothrombin times (15.8 s), and a reduced number of platelets (100 G/L). Protein C and Protein S activity was normal (28% and 48%, respectively). Troponin I was 202.8 ng/L, and the N-terminal prohormone of brain natriuretic peptide (NT-proBNP) was >35.000 pg/mL, suggesting myocardial damage and heart failure.

The neonate received two treatment doses of 4.5 milligrams of a low molecular weight heparin (LMWH) on the second day of life. Prolonged bleeding time from injection sites was observed. Coagulation tests showed activated partial thromboplastin time (APTT) above 400 s, decreased fibrinogen (1.61 G/L), and elevated anty-Xa activity (1.92 U/mL). Fresh frozen plasma was transfused, and normalization of the coagulation parameters was observed.

The neonate was transferred to the Cardiology Department on the 3rd day of life, where he received a continuous infusion of unfractionated heparin. However, due to the difficulties in maintaining appropriate APTT values and observed thrombocytopenia, the treatment was changed to LMWH. The activity of anti-Xa was monitored regularly. Moreover, antithrombin III was supplemented as its activity was reduced to 35%. Subsequent echocardiographic tests were performed to monitor the effects of the applied treatment. The echocardiography performed on the 9th and 12th day of life did not show the embolism, suggesting it had resorbed completely. The results of the follow-up abdominal ultrasound did not show any abnormalities. On the 12th day of life, the patient was discharged home in good condition.

## 3. Discussion

SARS-CoV-2 is a single-stranded ribonucleic acid (RNA) β-coronavirus. Using a specific host protease, transmembrane serine protease 2 (TMPRSS2) [14], it binds to the host cell receptor-angiotensin-converting enzyme 2 receptor (ACE2-R) with the major spike glycoprotein (S1) [15]. ACE2-R is expressed in various tissues and organs, e.g., the lungs, heart, intestine, muscles, liver, pancreas, or kidneys and on the epithelial cells of oral mucosa and the tongue [16,17]. Both arterial and vascular endothelium is characterized by high levels of ACE2-R expression as well [18]. The binding of the virus causes a decrease in the receptor activity, resulting in the accumulation of angiotensin II, which triggers intracellular signaling pathways (caspase 3, p83 MAPK, ROS, cytochrome C) and, subsequently, leads to vasoconstriction, increased oxidative stress, cellular damage, proinflammatory effect, and fibrosis [19]. Moreover, the replication of the virus inside the host cells may promote the immune response, releasing interferon-γ and interleukins: IL-1β and IL-6, which facilitates endothelial activation and inflammation [19,20].

Healthy endothelium is antithrombotic but might become prothrombotic when activated. COVID-19 infection determines endothelial activation by angiopoietin-2, a mediator stored in the Weibel–Palade body, which shows elevated circulating levels in COVID-19 and an association with the induction of procoagulant and proinflammatory reactions [21]. Endothelial activation promotes platelet recruitment through the secretion of the von Willebrand factor and expression of fibrinogen and P-selectin on the surface. Platelet aggregation might generate a deposition of platelet-rich clots in the lung microcirculation. This event is the key mechanism leading to respiratory failure. Furthermore, endothelial cells upregulate the expression of adhesion molecules: VCAM-1, ICAM-1, and E-selectin, which promote leukocyte adhesion and activation. The interaction of platelets and leukocytes facilitates the coagulation pathway and proinflammatory molecules secretion [20]. After the systemic activation of the coagulation and the development of disseminated microthrombosis, multiple organs will be affected.

Ackermann et al., in their study, presented results from autopsies performed on patients who died because of COVID-19. They examined their lungs and described that endothelial cells in the specimens were swollen, the intercellular junctions were disrupted, and there was a lack of contact with the basal membrane. The findings proved that infection with SARS-CoV-2 caused injury to the endothelium and can promote thromboembolism formation [22]. It was predicted that the injury of pulmonary endothelial cells contributed significantly to diffuse alveolar damage and the development of acute respiratory distress syndrome (ARDS) [23]. In another study, a post-mortem autopsy of severe COVID-19 patients confirmed diffuse alveolar damage and inflammatory infiltrations with hyaline membrane formation in the lung and, also, inflammation of the myocardium, focal pancreatitis, axon injury, glomerular microthrombosis, macrophage accumulation in the brain, and lymphocyte infiltrations of the liver [24].

The possible complications of SARS-CoV-2 infections are currently the subject of many studies. However, knowledge regarding the neonatal population is relatively scarce. During the COVID-19 pandemic, the prevalence of prothrombotic and cardiovascular complications increased. They occurred in about 9% of all adult patients [25], with up to 50% of those with severe manifestations [26]. These patients were more susceptible to developing deep vein thrombosis, arterial thrombosis, pulmonary embolisms, or intracatheter thrombosis [20], which were usually related to poorer prognosis and higher mortality rates [27]. However, among pediatric patients suffering from COVID-19, these complications were rather rare [28]. The incidence of thromboembolisms was lower in this group than in adults [29].

Schulze-Schiappacasse et al. published a case report of a 27-day-old neonate with a severe SARS-CoV-2 infection. At first, he presented with watery diarrhea and food refusal for 48 h, and, later, he developed pulmonary thrombosis. Despite the therapy with LMWH, the thrombus continued to grow. Therefore, the neonate required two courses of alteplase, which improved his clinical condition. Many factors could have contributed to the development of the disease, but SARS-CoV-2 infection might be treated as a condition promoting the thrombotic event [30]. However, to the best of our knowledge, no case report has been published where pulmonary thrombosis occurred in utero and caused circulatory failure in the fetus.

Multisystem inflammatory syndrome in neonates (MIS-N) is a syndrome similar to MIS-C, which has been well-described in pediatric patients. The reasons for neonates developing the syndrome are maternal infection and transplacental transfer of SARS-CoV2 antibodies or disease after birth [31]. Possible symptoms include increased CRP and cardiac enzymes, abnormal coagulation tests, cardiomegaly, hepatomegaly and splenomegaly, abnormal liver and kidney function tests, free peritoneal fluid, abnormalities in the brain, and low albumin levels. Compared to MIS-C, fever is not always observed. The outcome is favorable in most cases. However, the observed mortality rate can be up to 9.2% in neonates with MIS-N. The neonate presented in this case report had several MIS-N features such as elevated CRP, increased cardiac enzymes, cardiomegaly, free peritoneal fluid, hepatomegaly, abnormal liver tests, and low albumin level. However, he did not present with abnormalities in the brain, leukocytosis with lymphopenia, or hyponatremia, which are also common.

The molecular mechanisms of MIS-C and MIS-N have been the subject of many studies. SARS-CoV-2 infection preceding MIS-C is usually asymptomatic, but it appears to activate several immunological pathways. SARS-CoV-2 infection is believed to stimulate T-cells, which results in the stimulation of macrophages, monocytes, B-cells, and plasma cells. All of the immune mechanisms, along with the cytokine release (cytokine storm), lead to hyperinflammation and the development of MIS-C [32]. A reduced number of NK cells and lower NK cell degranulation was also identified as a possible factor in the immunopathogenesis of MIS-C [33].

Distinguishing MIS-C from other similar inflammatory syndromes remains challenging, given the lack of information about possible SARS-CoV-2 exposure in many cases. In order to facilitate the differential diagnosis, signatures of MIS-C were compared with severe COVID-19, Kawasaki disease, toxic shock syndrome, or hemophagocytic lymphohistiocytosis (HLH) [32,34]. The comparison studies aimed to identify a profile of inflammatory biomarkers that would be unique for MIS-C. The results indicated that MIS-C and Kawasaki have partially overlapping cytokine profiles, with elevated inflammatory markers such as IL-6, IL-18, IL-17a, or IFN-γ [35,36]. However, higher levels of IL-17a in Kawasaki disease might suggest different immunopathology. It has been proven that cytokine and chemokine profiles differed in severe COVID-19 and MIS-C. However, there is no consensus on MIS-C distinctive biomarkers [35,37,38]. MIS-C patients were characterized by higher expression of IL-6, higher levels of IFN-γ-induced chemokines (CXCL9 and CXCL10), and higher expression of IFN-γ in T-cells [33,39].

Comparing patients with MIS-C and with HLH, T-cell activation and TH1 cytokines were found in both groups but they differed in amplitude [34]. Hyperinflammation and cytokine storms were described in severe COVID-19 patients as well. According to the studies on COVID-19 complications, the cytokine storm might contribute to thromboembolism formation and multiorgan damage [20].

Thromboembolic complications have been described among the pediatric population both in COVID-19 patients and MIS-C [10,40,41]. Thromboembolisms occur more often among children suffering from MIS-C, with an incidence rate ranging from 1.4% to 6.5% [41]. There are several molecular mechanisms involved in thromboembolism formation. Hyperinflammation and cytokine storms contribute to endothelial dysfunction and hyperactivation of platelets. Moreover, activation of the complement described in MIS-C patients is suspected to increase the risk of thrombosis development.

Although MIS-C and COVID-19 are both unique risk factors, the pathogenesis of thromboembolism formation remains very complex and many factors play a role, including genetic mutations. There has been much research on various types of thrombophilia. It is known that factor V Leiden or prothrombin mutations increase the risk of venous thromboembolisms during SARS-CoV-2 infections [42,43]. PAI-1 gene mutation is a risk factor for myocardial infarctions and venous thromboembolism formation [44]. It may contribute to the severity of COVID-19 infection and lead to coagulopathy characterized by thrombi formation [45]. However, the impact of PAI-1 5G/4G polymorphism on COVID-19 severity has not been confirmed yet [27]. Moreover, the most frequent MTHFR gene polymorphisms C677T and A1298C had also been alleged to contribute to the more severe course of COVID-19. The possible impact of these polymorphisms is still being evaluated in the research studies [27]. However, according to the guidelines established by the American College of Medical Genetics in 2013 [46], the compound 677/1298 heterozygote polymorphism is unlikely to be an independent risk factor for thrombosis occurrence [47]. Given all of these examples, the genetic mutations of the presented neonate do not seem to be a leading factor contributing to pulmonary embolism formation. The possible causes of pulmonary thrombosis appear to be maternal SARS-CoV-2 infection and MIS-N.

The treatment of neonates with pulmonary thrombosis remains a challenge, as there is no consensus on the most favorable method. They include low molecular weight heparin, unfractionated heparin, thrombolytic therapy with alteplase, and catheter-based embolectomy [48]. Coagulation tests must be performed frequently to monitor the treatment. Decisions should be made carefully based on the extent of the thrombosis and modified as the patient is observed day by day to minimize the side effects of the treatment. More research regarding thrombosis treatment strategies is necessary. The usage of novel technologies such as artificial intelligence and machine learning has been described in the literature and might contribute to further search for possible therapies [49,50].

## 4. Conclusions

Given the positive result of IgG antibodies against SARS-CoV-2 and the mother’s infection in late pregnancy with symptoms suggesting COVID-19, we suspected that SARS-CoV-2 was a major factor associated with the formation of the pulmonary embolism in the presented newborn.

More research is necessary to evaluate the possible neonatal complications of maternal SARS-CoV-2 infection. In the described case, the neonate’s heart failure was diagnosed prenatally, which resulted in the admission to a 3rd reference-level hospital. A correct diagnosis was made, allowing for effective treatment. It is essential that pregnant women suffering from COVID-19 are monitored to detect possible abnormalities.
